# La varicelle périnatale: risques et prise en charge fœtale et néonatale

**DOI:** 10.11604/pamj.2017.28.233.8266

**Published:** 2017-11-15

**Authors:** Jihène Methlouthi, Nabiha Mahdhaoui, Manel Bellalah, Hedia Ayache, Sonia Nouri, Hassen Seboui

**Affiliations:** 1 Service de Néonatologie, CHU Farhat Hached, Sousse, Tunisie

**Keywords:** Varicelle, grossesse, nouveau-né, prévention, traitement, varicella, pregnancy, new-born, prevention, treatment

## Abstract

La survenue d'une varicelle clinique en cours de grossesse est rare, pouvant entrainer des risques maternels et fœtaux. La varicelle maternelle périnatale peut entrainer une varicelle néonatale potentiellement grave, notamment en cas d'éruptions maternelles survenant entre 5 jours avant et 2 jours après l'accouchement. Nous rapportons huit observations de nouveau-nés de mères ayant eu une varicelle en péri-partum dans le but de synthétiser, l'état actuel des connaissances sur le risque de ce virus et d'essayer d'élaborer un protocole de prise en charge. Il s'agit d'une étude descriptive menée au centre de maternité et de néonatologie de Sousse, sur une période de 10 ans. Huit nouveau-nés ont été inclus dans l'étude. Le diagnostic était fait avant l'accouchement chez 7 mères. Une seule femme avait développé sa varicelle 3 jours après l'accouchement. Cinq nouveau-nés étaient symptomatiques à l'admission. Ils avaient tous des lésions cutanées typiques de la varicelle, trois parmi eux avaient une atteinte respiratoire associée. La prise en charge était basée sur l'isolement des nouveau-nés, les soins locaux, et le traitement par Acyclovir. L'évolution était favorable dans tous les cas. La survenue d'une varicelle au cours de la grossesse reste possible dans les pays ou la vaccination n'est pas encore accessible à tous. Les risques de complications maternelles et fœtales qu'elle occasionne justifient une prise en charge spécifique et bien codifiée.

## Abstract (en)

The occurrence of clinical varicella during pregnancy is rare but it may pose maternal and fetal risks. Perinatal maternal varicella may result in potentially severe neonatal varicella, especially when maternal eruptions occur between 5 days before and 2 days after delivery. We report eight cases of newborns of mothers with varicella in the peri-partum period in order to synthesize the current state of knowledge on the risk of contracting virus as well as to develop treatment protocol. We conducted a descriptive study at the Maternity and Neonatology Center, Sousse, over a period of 10 years. Eight newborns were included in the study. Prenatal diagnosis was made in 7 mothers. Only a woman developed varicella 3 days after delivery. Five newborns were symptomatic on admission. All newborns had typical varicella skin lesions, three of them had respiratory distress associated. Treatment was based on newborn isolation, local skin care and Acyclovir therapy. Patients evolution was favorable. The occurrence of varicella infection during pregnancy remains possible in the countries where vaccination is still not accessible to all. The risk of maternal and fetal complications justifies specific and well codified treatment**.**

## Introduction

La varicelle est une infection strictement humaine très contagieuse, généralement bénigne, survenant dans 90% des cas avant l'âge de 15 ans. L'infection d'une femme enceinte non immunisée est une situation rare, pouvant l'exposer à différentes complications: varicelle grave de l'adulte, transmission materno-foetale avec varicelle congénitale en première moitié de grossesse, et infection néonatale sévère en cas d'infection maternelle en péri-partum. L'incidence de la varicelle congénitale suite à une varicelle maternelle avant 24 semaines d'aménorrhée (SA) est de l'ordre de 0,7 à 2%. En cas d'atteinte maternelle en péri-partum, le risque d'atteinte néonatale est de l'ordre de 20 à 50% avec un taux de mortalité de 20% parmi les nouveau-nés infectés [1]. Nous rapportons huit observations de nouveau-nés de mères ayant eu une varicelle en péri-partum dans le but de synthétiser, l'état actuel des connaissances sur le risque materno-fœtal de ce virus et d'essayer d'élaborer un protocole de prise en charge préventif et curatif des nouveau-nés à risque.

## Méthodes

Il s'agit d'une étude rétrospective menée sur une période de 10 ans allant du 1^er^ Juin 2005 au 31 Mai 2015. Nous avons inclus tous les nouveau-nés issus de mères ayant une varicelle périnatale et hospitalisés dans le service de néonatologie du Centre Hospitalo-Universitaire Farhat Hached de Sousse en Tunisie.

## Résultats

**Figure 1:**
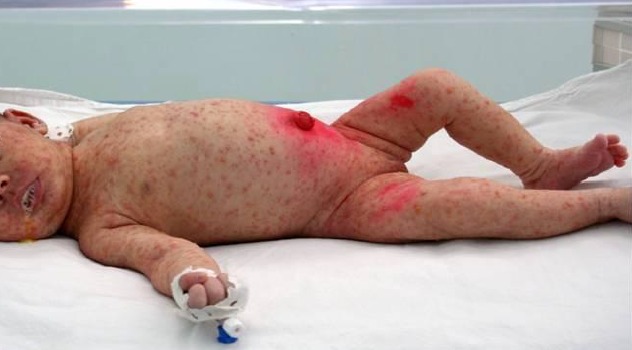
Photographie de l’observation n°2 mettant en évidence une éruption cutanée vésiculeuse évocatrice d’une varicella

**Figure 2:**
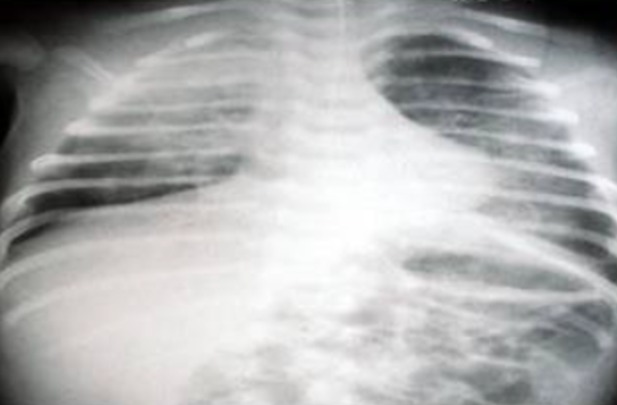
Radiographie du thorax de face de l’observation n°4 montrant une pneumopathie bilatérale (apicale droite et basale gauche)

Nous avons colligé 8 nouveau-nés issus de mères ayant eu une varicelle périnatale (Tableau 1). Le diagnostic était fait avant l'accouchement chez 7 mères avec un délai moyen de 3,2 jours et des extrêmes entre 1 et 5 jours. Une seule femme avait développé sa varicelle 3 jours après l'accouchement. Il s'agissait de 6 nouveau-nés à terme et 2 prématurés. Trois nouveau-nés étaient asymptomatiques à la naissance, hospitalisés d'emblé dans le service, les autres nouveau-nés étaient nés dans des maternités périphériques, ils étaient admis secondairement dans le service. Ils avaient tous des lésions cutanées typiques de la varicelle (Figure 1), trois parmi eux avaient une atteinte respiratoire associée (Figure 2). L'âge du début de la symptomatologie était entre le 5^ème^ et le 11^ème^ jour postnatal. La contamination par le virus de la varicelle était confirmée par PCR positive réalisée chez seulement deux nouveau-nés. La prise en charge était basée sur l'isolement des nouveau-nés, les soins locaux, et le traitement par Acyclovir. L'évolution était favorable dans tous les cas.

**Tableau 1:** Récapitulatif des huit observations (terme, délai entre varicelle maternelle et accouchement, signes cliniques, traitement et évolution)

	Terme	varicelle maternelle / accouchement	Signes cliniques+ Age	Traitement	Durée traitement	Durée hospitalisation	Evolution
Observation 1	A terme	J-1	Asymptomatique	Aciclovir (IV)	07 jours	08 jours	Favorable
Observation 2	A terme	J-5	Atteinte cutanée J 5 de vie	Aciclovir (IV)	07 jours	10 jours	Favorable
Observation 3	A terme	J-4	Atteinte cutanée J 11 de vie	Aciclovir (IV)	07 jours	10 jours	Favorable
Observation 4	A terme	J-3	-Atteinte cutanée à J 10 de vie + Pneumopathie	oxygénothérapie Aciclovir (IV)	07 jours	10 jours	Favorable
Observation 5	33 SA	J+2	Asymptomatique	Aciclovir (IV)	07 jours	28 jours	Favorable
Observation 6	A terme	J-3	Atteinte cutanée + DRNN à J6 de vie	Aciclovir (IV)	07 jours	09 jours	favorable
Observation 7	36 SA	J-2	Atteinte cutanée + DRNN à J7 de vie	Aciclovir (IV)	07 jours	12 jours	Favorable

## Discussion

La varicelle est une maladie bénigne de l'enfance, mais elle peut être dangereuse chez la femme enceinte, le fœtus, le nouveau-né et l'individu immuno-déficient. Le virus responsable est le virus de la varicelle et du zona (VZV). La fréquence de la varicelle chez la femme enceinte non immunisée est estimée entre 2 et 3 pour 1000 grossesses [1]. Elle l'expose à différentes complications: varicelle grave de l'adulte, varicelle congénitale (transmission materno-fœtale durant la première moitié de la grossesse) et infection néonatale sévère si l'infection maternelle survient en pré-partum ou en post partum précoce. La transmission de VZV de la mère au fœtus est la conséquence de la virémie maternelle avec un passage transplacentaire et possibles échanges de sang maternel et fœtal au moment de l'accouchement, voire beaucoup plus rarement, une contamination par voie ascendante a partir de la filière génitale. En postnatal, la transmission à un nouveau-né, non protégé par les anticorps maternels, peut avoir lieu par voie respiratoire et cutanée. L'adulte infecté peut être la mère mais aussi un tiers [2]. La varicelle maternelle au cours du 1^er^ ou du 2^ème^ trimestre de la grossesse expose le nouveau-né au risque d'embryopathie et donc le syndrome de la varicelle congénitale. Ce risque est de 0,4% avant 13 SA et de 2% entre 13 et 20 SA [3]. L'incidence de la varicelle congénitale est estimée à 1,6 cas pour 100 000 naissances. La majorité des cas rapportés concerne celle survenue avant le terme de 20 SA. Au-delà de ce terme, elle est exceptionnelle et il s'agit plutôt d'une varicelle néonatale. La période d'incubation chez la mère est caractérisée par deux phases de virémie, entre le 4^ème^ et le 6^ème^ jour et entre le 10^ème^ et 14^ème^ jour post exposition. La deuxième virémie semble plus marquée et le risque de passage transplacentaire du virus est plus important. Les anticorps anti-VZV apparaissent 10 à 20 jours après le contage et sont transmis au f'tus pour lui procurer une immunité passive. Le VZV a une forte affinité neurotrope et ses effets sur le système nerveux peuvent expliquer une grande partie des malformations. Après 20 SA le mécanisme est différent et on observe un tableau qui se rapproche de la varicelle néonatale [1]. La varicelle congénitale se caractérise par un spectre de malformations avec des lésions cutanées, neurologiques, oculaires et squelettiques (Tableau 2) [4]. Le diagnostic peut se faire en anténatal ou en postnatal. 76% des nouveau-nés présentent des lésions cutanées à type de cicatrice ou aplasie. L'atteinte neurologique arrive en deuxième position dans 60% des cas et inclut l'atrophie cérébrale, la microcéphalie, la paralysie des cordes vocales et les paralysies périphériques. L'atteinte oculaire sous forme de cataracte ou de choriorétinite est retrouvée chez 51% des nouveau-nés. L'atteinte squelettique et l'hypoplasie osseuse sont localisées, comme les atteintes cutanées, au niveau des dermatomes. Elles sont présentes dans 49% des cas. Le retard de croissance intra-utérin est fréquemment associé. Les calcifications hépatiques sont rares et ne sont pas spécifiques du virus de la varicelle [5]. Le diagnostic de l'infection fœtale repose sur la détection de l'ADN viral dans le liquide amniotique dans un prélèvement réalisé après 22 SA, et au moins 5 semaines après le début de l'éruption maternelle.

**Tableau 2:** Principaux signes cliniques de la varicelle congénitale dans la littérature

Symptômes	Nombre d’enfants (*n* = 124)	
	*n*	%
Lésions cutanées (cicatrices ou aplasie)	89	72
Atteinte neurologique (atrophie cérébrale, microcéphalie, paralysie des cordes vocales, paralysies périphériques, convulsions…)	77	62
Atteinte oculaire (microphtalmie, enophtalmie, choriorétinite, cataracte, nystagmus, atrophie optique…)	65	52
Atteinte squelettique et hypoplasie osseuse	55	44
Retard de croissance intra-utérin	28	23
Anomalies digestives	25	20
Hypoplasie musculaire	24	19
Anomalies génito-urinaires	15	12
Atteinte des organes internes	14	11
Retard du développement	13	10
Anomalies du système cardio-vasculaire	9	7
AUTRES	9	7

Le risque de varicelle néonatale est présent lorsque l'infection maternelle survient dans les trois semaines qui précédent l'accouchement. Ce risque dépend du délai entre l'éruption de la mère et l'accouchement. La gravité est maximale lorsque d'éruption maternelle survient cinq jours avant (J-5) et deux jours après (J+2) l'accouchement puisque le nouveau-né est infecté par voie transplacentaire sans passage des anticorps maternels. Le risque de varicelle néonatale sévère dans cette période est de 20 à 50% [1]. Le nouveau-né va présenter une infection disséminée avec fièvre, éruption hémorragique, atteinte pulmonaire et hépatique. Le pronostic spontané est grave avec une mortalité néonatale de 30% [1,5]. Dans notre série, la varicelle maternelle était survenue entre 1 et 5 jours avant l'accouchement dans 7 cas avec un délai moyen de 3,2 jours. Une seule femme a développé sa maladie 3 jours après l'accouchement. Trois nouveau-nés étaient asymptomatiques à la naissance, hospitalisés d'emblé dans le service, ce sont ceux qui étaient nés à la maternité centrale, les autres nouveau-nés (5 cas) étaient nés dans des maternités périphériques. La traduction clinique de cette infection est directement corrélée au passage placentaire des anticorps (Ac) maternels, qui débute en moyenne 3 jours après le début de l'éruption, pour être complet 1 semaine après le début de l'éruption. Si l'éruption maternelle survient plus d'une semaine avant l'accouchement, le virus et les anticorps maternels traversent la barrière placentaire. Dans ce cas, seuls 23% des nouveau- nés seront symptomatiques. Si l'éruption maternelle débute entre j-5 à j +2 par rapport à l'accouchement, le virus sera transmis au nouveau-né avec un risque maximal de varicelle néonatale sévère, dont la mortalité était évaluée autour de 30% avant l'ère des immunoglobulines spécifiques [5]. L'incubation est plus courte, avec un délai de 12 jours entre l'éruption maternelle et l'éruption néonatale expliquée par une inoculation directement intraveineuse. Une varicelle survenant à moins de 12 jours de vie traduit une acquisition périnatale, tandis qu'une varicelle survenant au-delà de ce délai traduit une acquisition post-natale [1]. L'âge du début des manifestations cliniques chez nos cinq malades symptomatiques était entre J5 et J11 de vie. Ils avaient tous des lésions cutanées typiques de la varicelle, trois parmi eux avaient une atteinte respiratoire associée. La varicelle maternelle s'était révélée entre J-2 et J-5. Le diagnostic de la varicelle néonatale est essentiellement clinique. Il est confirmé par la sérologie qui est largement utilisée. La présence d'IgM est le reflet d'une infection récente il faut se méfier des réactions croisées avec l'herpès simplex virus. La détection du VZV par amplification du génome (PCR) sur les prélèvements au niveau des vésicules ou les secrétions trachéales est une méthode plus sensible et spécifique [3]. Le traitement étiologique repose sur l'aciclovir et le valaciclovir. L'aciclovir traverse la barrière placentaire, et les concentrations sériques fœtales atteignent 15 à 20% des concentrations maternelles. Le valaciclovir est la pro-drogue de l'aciclovir, mieux absorbée par voie orale et traverse également la barrière placentaire [1]. Le traitement maternel par Aciclovir ou Valaciclovir réduit la morbidité et la mortalité de la varicelle chez la mère notamment lorsqu'il est administré dans les 24 heures suivant le début de l'éruption [6].

**Figure 3:**
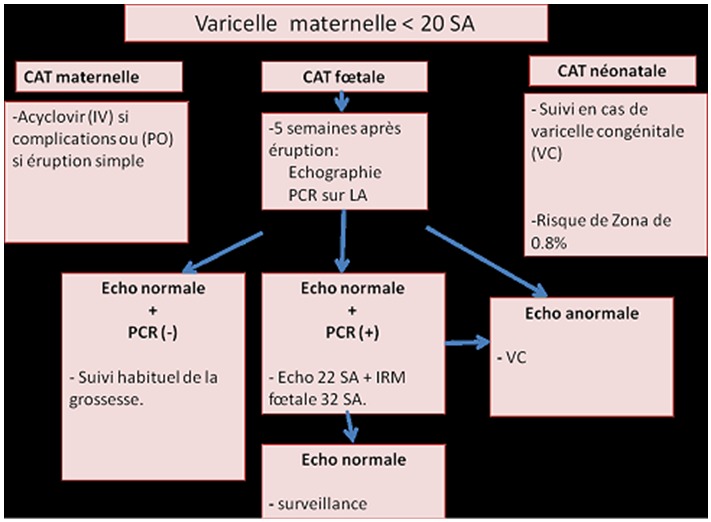
Diagramme résumant la conduite à tenir devant une varicelle maternelle survenant avant 20 SA

**Figure 4:**
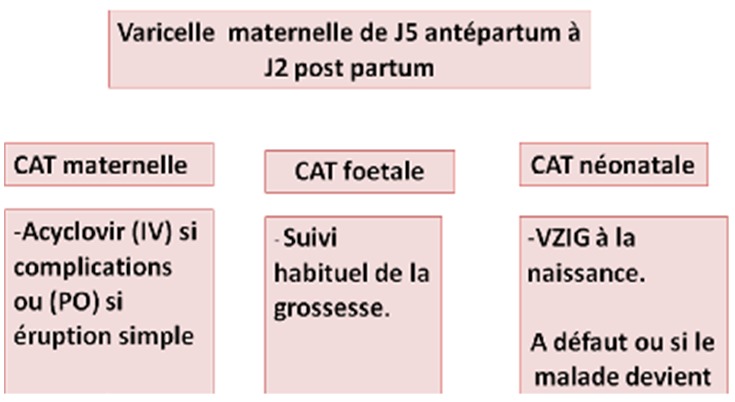
Diagramme résumant la conduite à tenir devant une varicelle maternelle survenant entre J5 antépartum et J2 postpartum

En cas de varicelle maternelle avant 20 SA (Figure 3), une surveillance échographique est pratiquée au fœtus associée à une recherche virale dans le liquide amniotique par PCR. La présence d'anomalies échographiques typiques oriente vers une varicelle congénitale et pourrait indiquer une interruption médicale de la grossesse (IMG) en concertation avec la famille et une équipe multidisciplinaire. Une PCR VZV positive sans lésions échographiques oriente vers un risque faible de varicelle congénitale et ne permet à elle seule d'indiquer une IMG, il faut continuer à surveiller le fœtus moyennant des échographies et une IRM fœtale. En cas de varicelle maternelle en fin de grossesse il vaut mieux retarder l'accouchement d'une semaine afin que le nouveau-né bénéficie des anticorps maternels. La prise en charge des nouveau-nés est actuellement bien codifiée et dépend du moment de la contamination. Dans la varicelle congénitale les nouveau-nés n'ont habituellement pas de maladie active mais de lésions cicatricielles. Théoriquement il n'y a pas d'indication à mettre en place un traitement anti-viral, mais des études ont montré un bénéfice sur la progression des lésions ophtalmologiques et neurologiques de la varicelle congénitale [2]. En cas de varicelle néonatale, tous les nouveau-nés atteints, pendant les 15 premiers jours de leur vie, doivent être traités par Aciclovir intra-veineux pendant 7 jours à la dose de 20mg/kg toutes les 8 heures. Ce traitement fait disparaitre la mortalité de la varicelle néonatale et réduit les séquelles. La mère et son enfant doivent être isolés des autres personnes. L'allaitement maternel est fortement conseillé quelque soit le statut immunologique de la mère [1]. En cas de fort risque d'atteinte fœtale suite à une varicelle maternelle 5 jours avant et deux jours après l'accouchement (Figure 4), un traitement préventif par immunoglobulines spécifiques du virus de la varicelle (VZIG) est indiqué chez les nouveau-nés asymptomatiques, associé à l'acyclovir en cas d'apparition des symptômes. En l'absence de VZIG, comme c'est le cas en Tunisie, le traitement par Aciclovir est mis en place dès l'accouchement pour une durée de 5 à 7 jours même en l'absence de signes cliniques chez le nouveau-né [7]. Cette conduite n'a été adoptée que chez 3 cas de notre série. Ce sont les nouveau-nés accouchés dans notre maternité. Ils étaient tous asymptomatiques et la contamination par le virus de la varicelle était confirmée par PCR positive chez deux parmi eux. La prévention primaire de la varicelle passe par la vaccination. Le vaccin vivant atténué (Varilrix^®^ ou Varivax^®^) est indiqué en dehors de la grossesse chez toute femme en âge de procréer et sans antécédent de varicelle clinique. Le schéma vaccinal repose sur l'administration de 2 doses espacées de 4 à 8 semaines ou de 6 à 10 semaines, selon le vaccin utilisé. Une contraception est recommandée dans le mois suivant la vaccination. La vaccination post-exposition, administrée dans les 3 à 5 jours suivant le contage, prévient à 100% la varicelle grave et réduit de 67 à 90% l'incidence de varicelle chez les sujets exposés. Toute vaccination anti-varicelleuse est cependant contre-indiquée chez la femme enceinte en raison du risque théorique de transmission verticale du virus vaccinal. La vaccination est en revanche possible en post-partum, y compris pendant l'allaitement. Le virus vaccinal ne semble pas passer dans le lait maternel, d'après les résultats d'une étude menée sur 217 échantillons de lait prélevés chez 12 femmes contaminées [8]. En l'absence du vaccin, comme c'est le cas de la Tunisie, la prévention de la varicelle périnatale est basée sur l'éviction du contage des femmes enceintes non immunisées avec les personnes contagieuses. Les immunoglobulines spécifiques du virus de la varicelle (VZIG) peuvent être indiquées en prévention secondaire. Elles sont commercialisées aux États-Unis depuis décembre 2012 sous le nom de VariZIG^®^, disponibles en autorisation temporaire d'utilisation (ATU) en France sous le nom de Varitect^®^ mais non disponibles en Tunisie.

**Figure 5:**
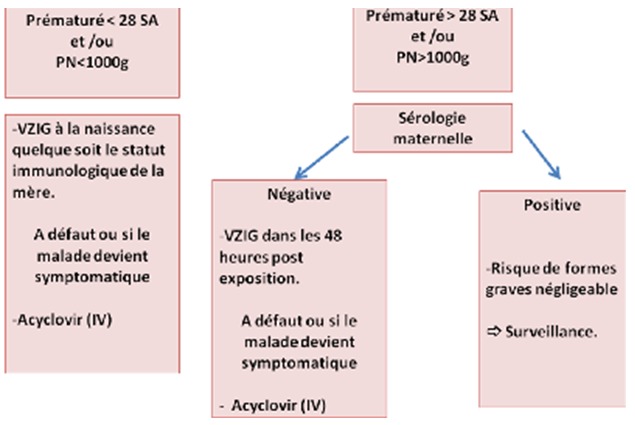
Diagramme résumant la conduite à tenir devant un contage néonatal durant les 28 premiers jours de vie

Il est important de connaitre les situations à risque de contagiosité pour les personnes séronégatives. C'est principalement un contage intra-familial, un contact physique proche avec une personne contagieuse pendant plus de 5min ou la présence dans la même pièce pendant plus d'une heure. Une personne est contagieuse 48 heures avant le début de l'éruption et 72h après la disparition de la dernière lésion cutanée [2]. Les immunoglobulines spécifiques permettent de diminuer la maladie donc de réduire le risque de transmission fœtale. Elles réduisent de 70 à 30% la fréquence des varicelles post-exposition. Dans le large travail prospectif d'Enders et al [9], aucune varicelle congénitale n'a été identifiée parmi les 97 femmes exposées traitées par immunoglobulines spécifiques. Selon Pastuszak et al [6] un seul cas de varicelle congénitale a été identifié chez une mère traitée par immunoglobulines parmi les 106 femmes de leur cohorte ayant contracté une varicelle avant 21 SA. Les VZIG, sont indiqués, le plus précocement possible, après la contamination d'une femme enceinte sans antécédents de varicelle. Le traitement est administré par voie intraveineuse (IV) ou intramusculaire (IM) à la dose de 12,5 UI/Kg avec un maximum de 625 UI. Ces VZIG ne doivent pas être administrés en cas de signes cliniques de varicelle. L'usage des immunoglobulines était limité aux expositions récentes, dans les 96 heures suivant le contage. Aux États-Unis, deux mises à jour récentes, une de la Food and Drug Administration (FDA) en 2012 [10], une des Centers for Disease Control and Prevention (CDC) en 2013, autorisent désormais l'usage des immunoglobulines spécifiques jusqu'à 10 jours après le contage. En cas de problème de procuration des immunoglobulines comme c'est le cas en Tunisie, ou en cas de contage dépassant les 10 jours, un traitement maternel par Aciclovir ou valaciclovir pourrait être recommandé. Administré 9 à 11 jours après le contage, il réduit de 77 à 7% ou de 100 à 16% [7,10] le taux de varicelle clinique chez l'enfant immunocompétent. Les nouveau-nés à risque de développer une varicelle néonatale peuvent aussi recevoir le VZIG. Il s'agit des nouveau-nés dont la mère a eu une varicelle 5 jours avant et 2 jours après l'accouchement, les nouveau-nés de mères séronégatives ayant eu un contage de varicelle durant les 18 premiers jours de vie ou les prématurés de moins de 28 SA et les nouveau-né avec un poids de naissance de moins de 1000 g ayant eu un contage durant les 28 premiers jours de vie, et ceci quelque soit le statut immunologique de la mère, vu qu'ils n'ont pas eu le temps d'avoir les anticorps maternels (Figure 5). La dose est de 0,5 ml/Kg en IM ou IV. Ce traitement ne diminue pas le risque de la maladie mais il réduit la gravité de l'atteinte [7].

## Conclusion

Le risque de varicelle congénitale est faible mais les conséquences peuvent être sévères. Il est important de mettre en place un protocole de dépistage anténatal fiable se basant sur une surveillance échographique et d'une PCR sur liquide amniotique. Seules les complications néonatales peuvent être prévenues par l'administration précoce de VZIG et la mise en place d'un traitement anti-viral selon les indications. Enfin la prévention passe essentiellement par l'éviction d'un contage et si possible la vaccination de toute femme non immunisée en âge de procréation exposée à un risque de contamination.

### Etat des connaissances actuelles sur le sujet

- La varicelle néonatale est rare mais grave notamment chez les nouveau-nés issus de mère ayant contracté la varicelle 5 j avant ou 2 jours après l'accouchement;
- Ces nouveau-nés à risque doivent recevoir des immunoglobulines spécifiques anti varicelle dès l'accouchement et avant qu'ils deviennent symptomatiques.

### Contribution de notre étude à la connaissance

- Dans les pays ne disposant pas des immunoglobulines spécifiques (VZIG), comme le notre, les nouveau-nés à risque doivent être hospitalisés et recevoir de l'aciclovir en intra veineux;
- D'après notre série, les trois nouveau-nés asymptomatiques ont bien évolué après l'application de ce protocole de prise en charge adapté à nos conditions.

## Conflits d’intérêts

Les auteurs ne déclarent aucun conflits d’'intérêts.
